# Plasmacytomas: many faces of one disease, or many diseases with one face?

**DOI:** 10.18632/oncotarget.26557

**Published:** 2019-01-08

**Authors:** Gaurav Goyal, Wilson I. Gonsalves

**Affiliations:** Gaurav Goyal: Division of Hematology, Mayo Clinic, Rochester, MN, United States

**Keywords:** plasmacytoma, radiation, myeloma, outcomes

**Figure 1:**
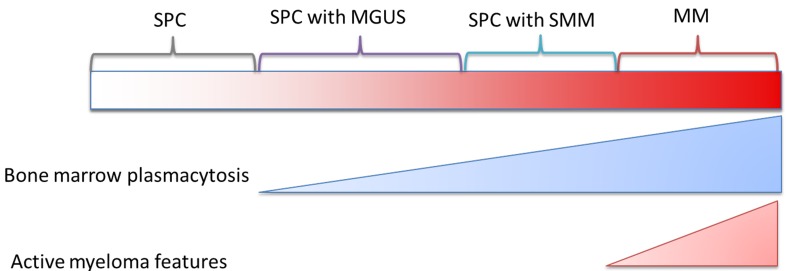
Theoretical continuum of progression of solitary plasmacytoma to multiple myeloma SPC: solitary plasmacytoma; MGUS: monoclonal gammopathy of undetermined significance SMM: smoldering multiple myeloma; MM: multiple myeloma.

Plasmacytomas are uncommon neoplasms of plasma cells that may arise from within or outside the bone marrow. They can either be solitary or multiple, the former called as solitary plasmacytoma (SPC). Based on location of the tumor, these can be classified as plasmacytomas of the bone (P-bone) or extramedullary plasmacytomas (P-EM). Despite numerous advances in deciphering the biology and management of multiple myeloma (MM), our understanding of plasmacytomas has lagged behind, partly due to their rarity and partly due to the heterogeneity of clinical manifestations. Nevertheless, our general understanding of the clinical spectrum of plasma cell neoplasms ranging from SPC to MM can be visualized in Figure 1. The reported outcomes of plasmacytoma are variable as well, with the highest risk of mortality resulting from its progression to MM [1, 2]. Most treatment recommendations for a plasmacytoma involve the use of radiation therapy, with or without surgical resection. However, these recommendations are based on expert opinion and not derived from rigorous prospective studies. In a rare disease such as plasmacytoma where prospective studies are not feasible, we often have to rely on retrospective data to answer key questions regarding clinical manifestations, treatment and outcomes.

In our recent work, we sought to answer some of these questions using the National Cancer Data Base (NCDB), which is a hospital-based database consisting of about 70% newly diagnosed cancer cases within the United States. The major strength of NCDB lies in the sheer volume of patients reported to this registry, which makes it ideal to study rare cancers such as plasmacytoma. In our recent report on plasmacytoma using NCDB, we focused on a pure cohort of patients with a plasmacytoma alone by excluding patients with reported bone marrow involvement [3]. We analyzed a large cohort of 5,056 patients with a plasmacytoma, of which majority (70%) were P-bone. Among the P-EM, most common site of disease was upper aerodigestive tract (45%), which is similar to other studies, where it has sometimes been alluded to as head/neck or pharynx [1, 2]. Our study demonstrated that P-EM had significantly better median overall survival (OS) as compared to P-bone (132 *vs*. 85 months). Among the P-EM group, the median OS for connective and soft tissue disease was worse than the rest (82 *vs*. 148 months) and almost similar to P-bone. On multivariable analysis, P-bone and age ≥ 65 years were found to be independent negative prognostic factors.

Additionally, we utilized a non-parametric machine learning model (recursive partitioning) to sequentially assess variables of importance that are associated independently with OS as an outcome [3]. This was performed on a cohort of 3,905 patients where complete radiation therapy data were available. In accordance with expert guidelines, we found the median dose of radiation therapy received to be 45 Gy. Among this cohort, radiation dose of <40 Gy was found to be an independent negative prognostic factor. Hence, if radiation therapy is being considered for plasmacytoma, our results confirm the existing guidelines recommending a dose of ≥40 Gy. Our analysis also showed that receipt of surgery and adjuvant radiation therapy was associated with improved OS as compared to radiation monotherapy [3]. The majority of patients who underwent surgery with adjuvant radiation had involvement of central nervous system and upper aerodigestive tract. In previous studies of P-EM, this combined therapeutic approach has been found to be associated with improved local control especially among patients with head and neck disease as well [4, 5]. In a recent study from the Surveillance, Epidemiology and End Results database, surgery and adjuvant radiation was also found to be associated with a superior OS among patients with P-bone involving the axial skeleton [2]. Hence, our results in conjunction with existing studies show that there may be value in obtaining surgical evaluation to assess complete resectability, especially with plasmacytomas involving the head, neck and axial skeleton.

Although our study provides insight into crucial patient- and treatment-related prognostic factors for OS, much more work needs to be undertaken to better identify robust risk factors and the molecular underpinnings for the progression of SPC to MM despite adequate initial therapy. Some known risk factors of progression from SPC to MM after initial therapy include increasing levels of angiogenesis within the plasmacytoma [6], persistence of circulating paraproteins [7], presence of bone marrow plasmacytosis, and increased FDG uptake in the plasmacytoma [8]. There is also evidence to suggest that the plasma cells of true SPC that are possibly cured with definitive local therapy such as radiation with or without surgery may have different expression of cell adhesion molecules and chemokines than the “neoplastic” plasma cells of MM, leading to less colonization of the former in the bone marrow and less likelihood of developing MM [9]. However, it is important to recognize that despite MM being almost always preceded by monoclonal gammopathy of undetermined significance [10], 40-60% of patients with a plasmacytoma do not demonstrate clonal bone marrow plasma cells or a measurable M-protein at diagnosis [1, 8]. Finally, current clinical trials are underway that assess the use of limited duration adjuvant chemotherapy for the management of SPCs after initial definitive local therapy. Being able to precisely identify those patients with SPC who are very likely to develop MM despite definitive local therapy could help us provide additional therapy in an adjuvant fashion and possibly improve survival outcomes.
